# Supplementary material for: Reliability assessment model for multiple stress factors accelerated degradation test using a Wiener process with random effects
Source: PLoS One. 2025 Jun 10;20(6):e0325117. doi: 10.1371/journal.pone.0325117 (PMC12151484; doi:10.1371/journal.pone.0325117)
Supplement: Attachment — Submitted filename: Response to reviewers.pdf [file pone.0325117.s001.pdf]

Response to Reviewer Comments on PONE-D-24-52301

Reliability assessment model for multiple stress factors accelerated degradation test using a Wiener process with random effects

Jiayin Tang[3]

[3] Department of Statistics, School of Mathematics, Southwest Jiaotong University, Chengdu, Sichuan, China

Dear Editor and Reviewers:

Thank you for your letter and for the insightful comments provided by the reviewer regarding our manuscript (PONE-D-24-52301). These comments are invaluable and greatly assist in enhancing the quality of our manuscript. We have thoroughly considered all the comments and have made corresponding corrections for each. We have carefully considered all of the feedback, our responses to the reviewers' comments are outlined below:

Response to Reviewer 1

Comment 1: Given the complexity of the model parameters, it is suggested that the authors explore the feasibility of other estimation methods (such as Bayesian methods) and compare the advantages and disadvantages of different methods.

Response 1: We sincerely thank the reviewer for the valuable suggestion to explore alternative estimation methods, such as Bayesian approaches, and to compare their respective advantages and disadvantages. In our study, we selected the current estimation method for its computational efficiency and proven effectiveness in addressing the specific characteristics of our data. This method has been widely utilized in similar contexts and has yielded reliable results in our preliminary analyses.

We acknowledge that Bayesian methods could offer significant advantages, particularly in terms of incorporating prior knowledge and addressing uncertainty. However, their implementation may necessitate additional computational resources and careful consideration of prior distributions, which could introduce subjectivity. Given the complexity of the model parameters, we agree that exploring Bayesian methods could provide valuable insights. We plan to conduct a comparative study in future work to evaluate the performance of various estimation methods, including Bayesian approaches, and assess their respective advantages and disadvantages within our specific context.

We appreciate the reviewer’s suggestion and will consider it a key direction for future research. We believe that such a comparative analysis could strengthen the robustness and generalizability of our findings.

Comment 2: The model mentioned in the paper is specific to the linear Wiener process. It is recommended that the authors consider extending the model to the nonlinear case to handle more complex degradation patterns.

Response 2: We appreciate the reviewer’s suggestion and believe that extending the model to the nonlinear case could significantly broaden the scope of our work. We will incorporate this direction into our future research agenda.

Comment 3: When constructing confidence intervals, the authors adopted the Bootstrap method. It is suggested that the authors discuss other methods for constructing confidence intervals (such as asymptotic methods) and compare the accuracy and efficiency of different methods.

Response 3: Thank you for your excellent comment. We believe that exploring alternative methods for constructing confidence intervals could significantly enhance the methodological depth of our work. While the Bootstrap method was chosen for its flexibility and robustness in our current study, in future research, we will focus on studying the generalized confidence intervals and Bayesian credible intervals for the model parameters and incorporate a comparison of different methods to provide a more comprehensive evaluation of their performance.

Response to Reviewer 2

Comment 1: The paper is well written. However, the following concerns should be addressed. The authors should compare the proposed models with other accelerated degradation models with multiple stress factors.

Response 1: Thanks so much for your constructive comment. In the simulation study and numerical example, the proposed model was compared with the linear random effects multi-stress Wiener accelerated degradation model that does not account for measurement errors. The results indicates that the proposed model exhibits good robustness.

Response to Reviewer 3

Comment 1: The main contribution of this paper is not prominently marked. Please summarize the innovations in the Introduction.

Response 1: We sincerely thank the reviewer for their valuable feedback and for pointing out the need to more prominently highlight the main contributions of our work. We appreciate the opportunity to clarify and emphasize the innovations of our study.

In the revised manuscript, the main contributions of this paper are introduced at the end of the fourth paragraph of the introduction.

Comment 2: How are the parameters of the Wiener process determined and verified?

Response 2: Thank you for your careful comment. The maximum likelihood estimates of the model parameters are derived using the profile likelihood approach. We believe that the methods used for determining and verifying the Wiener process parameters are robust and well-justified.

Comment 3: There are many models of random processes. Why is the Wiener process suitable for this case?

Response 3: Thank you for the valuable comments. The Wiener process is suitable for this study primarily because of its significant advantages in describing random processes and uncertainties in continuous time. Specifically, the increments of the Wiener process are independent and normally distributed, which allows it to naturally capture random fluctuations or noise. This is particularly well-supported in the context of the phenomenon we are studying, where the assumption of Gaussian increments holds. Besides, the Wiener process is continuous and unbiased, with variance increasing linearly over time, which aligns with the core characteristics of the random phenomena we aim to model. Although other accelerated degradation models based on random processes, such as the Poisson process, may be suitable for modeling discrete events, they are not appropriate

for capturing the continuous dynamics in our study. Therefore, the Wiener process with random effects is a reasonable and effective choice, accurately reflecting the randomness and uncertainty of the phenomenon.

Comment 4: Recent reliability assessment related work could be referred to, Artificial intelligence enhanced fault prediction with industrial incomplete information, Data-model-linked remaining useful life prediction method with small sample data: A case of subsea valve, Remaining useful life estimation of structure systems under the influence of multiple causes: Subsea pipelines as a case study, Remaining useful life re-prediction methodology based on Wiener process: Subsea Christmas tree system as a case study.

Response 4: We sincerely thank the reviewer for highlighting recent and highly relevant studies in the field of reliability assessment. The studies mentioned by the reviewer provide valuable insights into advanced reliability assessment methods, particularly in industrial applications. In our original manuscript, we focused on foundational and widely recognized literature to establish the theoretical framework for our approach. However, we acknowledge that incorporating these recent works would enhance the context and relevance of our study.

Comment 5: I think there is still a lot of room for improvement in the conclusion part, especially the summary of the work done needs to be further strengthened.

Response 5: We sincerely thank the reviewer for pointing out the need to improve the conclusion section of our manuscript. We agree that the conclusion is a critical part of the paper, and we have carefully revised it to provide a more comprehensive and impactful summary of our work.

Response to Reviewer 4

Comment 1: the degradation model is too usual without much new contribution, a more realistic and complex models should be considered and the engineering background is needed. The engineering mechanism for the failure process is very important for reliability research. Thus, a novel degradation is needed first and the parameter estimation process could be much brief. For example, considering the shock process, multiple degradation indexes, etc.

Response 1: We sincerely appreciate the reviewer's valuable comments and suggestions. We acknowledge that the degradation model presented in the original manuscript is relatively conventional and may not fully capture the complexity of real-world engineering scenarios. We agree that a more realistic and complex model is needed to better reflect the engineering context and failure mechanisms. We plan to consider complex models as our future work. This is the basis.

Comment 2: What is the application value of the research and the results. A more management view is needed for the conclusion.

Response 2: We sincerely appreciate the reviewer's valuable feedback regarding the application value of our research. Our research has significant application value in the field of reliability theory and engineering. The proposed model and results can be directly applied to reliability analysis based on Wiener accelerated degradation model, enabling engineers and managers to make more informed decisions. To further illustrate the application value, we have included a case study based on a numerical example. The results demonstrate how the proposed approach can be implemented in real-world scenarios, such as reliability assessment for highly reliable products.

Response to Reviewer 5

Comment 1: More discussions should be provided to prove creativity of the present work in the Introduction part.

Response 1: Thank you for the valuable suggestions regarding the introduction. We fully understand the reviewer's expectation to highlight the creativity of our work. To better emphasize the innovation of our study, we will add more comparisons between the current research and existing literature in the introduction, and provide a detailed explanation of the unique aspects of our proposed method or theoretical framework in addressing the specific problem.

Comment 2: A nomenclature is suggested since there are too many abbreviations used.

Response 2: Thank you for the valuable suggestion regarding the use of abbreviations. We understand that the frequent use of abbreviations may affect the readability and clarity of the paper. To address this issue, we will follow the reviewer's suggestion and include a nomenclature section in the paper, listing all abbreviations along with their corresponding definitions. We believe this will help improve the readability of the paper and ensure that readers can more easily understand our research.

Comment 3: Reasonability and generality of the basic assumptions should be clearly discussed, especial A2 and A3.

Response 3: Thank you for the valuable suggestion regarding the discussion of the reasonability and generality of the assumptions. We fully understand the reviewer's concerns about assumptions A2 and A3, particularly regarding their impact on the research results.

A2 is an assumption regarding the consistency of the degradation mechanism. The scale parameter σ of the Wiener process reflects the 'amplitude' or volatility of the stochastic process. Therefore, to ensure the consistency of the degradation mechanism, a reasonable assumption is that the scale parameters under different test groups are statistically equal.

In practical cases, there exists substantial unit-to-unit variability among degradation processes of different individuals, mainly because of the differences in materials and production conditions. Some studies have shown that such heterogeneities can be well captured by incorporating random effects into the degradation process. Therefore, A3 assume that the drift parameter β to be a random variable, accounting for the unit-to-unit variability.

Comment 4: Transformed variables x_{iq} in A3 was referred to as accelerating variables in the beginning of Section 2.2, please have a check and uniform them.

Response 4: We greatly appreciate the reviewer careful comment. In the revised manuscript, $x_{iq}, i = 1,2,\ldots,l, \quad q = 1,2,\ldots,p$, denotes the i-th stress loading level of the q-th stress factor (i.e., accelerating variable).

Comment 5: “,” is suggested for notations with subscripts, for example, “t_ijk” shoud be “t_i,j,k” since i, j, k might take values more than 10.

Response 5: Thank you for your valuable suggestion regarding the notation format. We understand that using commas in subscripts, such as “$t_{i,j,k}$” can help avoid ambiguity when indices exceed single digits. However, in our manuscript, the indices *i*, *j*, and *k* are consistently defined within a range that does not exceed single-digit values. Therefore, the current notation “t_{ijk}” does not lead to any confusion in our context.

Comment 6: There are many formulae and numbers in this manuscript, please check them carefully to make sure they are all correct without any typos and mistakes.

Response 6: Thank you for your careful review and valuable suggestion. We fully agree with the importance of ensuring the accuracy of all formulae and numerical values in the manuscript. In response to your comment, we have thoroughly reviewed all the equations, calculations, and numerical data presented in the manuscript to ensure there are no typos or mistakes. We have also double-checked the derivations and cross-verified the results to confirm their correctness. If there are any specific parts of the manuscript that you would like us to revisit or clarify further, please kindly point them out, and we will address them promptly.

Comment 7: In the reference list, please list all authors instead of using “et al.”, and the using of spaces should also be carefully uniformed.

Response 7: Thank you for your careful review and valuable suggestions. We appreciate your attention to detail in improving the quality of our manuscript. In response to your comment, we have revised the reference list to include all authors instead of using “et al.” In addition, we have carefully checked and unified the use of spaces throughout the reference list to ensure consistency.
