# Supplementary material for: Reliability assessment model for multiple stress factors accelerated degradation test using a Wiener process with random effects
Source: PLoS One. 2025 Jun 10;20(6):e0325117. doi: 10.1371/journal.pone.0325117 (PMC12151484; doi:10.1371/journal.pone.0325117)
Supplement: Attachment — Submitted filename: Response_to_reviewers_auresp_2.pdf [file pone.0325117.s002.pdf]

Response to Reviewer Comments on PONE-D-24-52301

Reliability assessment model for multiple stress factors accelerated degradation test using a Wiener process with random effects

Jiayin Tang[3]

[3] Department of Statistics, School of Mathematics, Southwest Jiaotong University, Chengdu, Sichuan, China

Dear Editor and Reviewers:

Thank you for your letter and for the careful comments proposed by the reviewer regarding our revised manuscript (PONE-D-24-52301R1). We have further revised the comments still raised by the reviewers. We have carefully considered all of the feedback, our responses to the reviewers' comments are outlined below:

Response to Reviewers

Reviewer #1: The authors have adequately addressed my comments. I also checked the authors' response to other reviewers' comments. The revision looks well prepared.

Reviewer #2: (No comments)

Reviewer #3: The author has addressed most of the concerns, but the main contribution of this article is still unclear. It is suggested that the author strengthen the introduction section. This article can be accepted after minor revisions.

Response： We sincerely thank the reviewer for the valuable suggestion to emphasize the main contribution of the paper. We have accepted suggestion and summarized the main contributions of the paper based on the opinions of the reviewers; Added specific details to the second to last paragraph of the introduction section on page 3. Correspondingly, we have also made corresponding modifications in the conclusion section. And highlight with blue .

Reviewer #5: The manuscript is now fine with me since previous comments of the revieweres have been properly addressed.
